# Actions of Brain-Derived Neurotrophin Factor in the Neurogenesis and Neuronal Function, and Its Involvement in the Pathophysiology of Brain Diseases

**DOI:** 10.3390/ijms19113650

**Published:** 2018-11-19

**Authors:** Tadahiro Numakawa, Haruki Odaka, Naoki Adachi

**Affiliations:** 1 Department of Cell Modulation, Institute of Molecular Embryology and Genetics, Kumamoto University, Kumamoto 860-0811, Japan; harukiodaka@gmail.com; 2 Department of Mental Disorder Research, National Institute of Neuroscience, National Center of Neurology and Psychiatry (NCNP), Tokyo 187-8551, Japan; 3 Department of Biomedical Chemistry, School of Science and Technology, Kwansei Gakuin University, Sanda city, Hyogo 669-1337, Japan; naookle@gmail.com

**Keywords:** brain-derived neurotrophic factor, neurogenesis, neurodegeneration, intracellular transport

## Abstract

It is well known that brain-derived neurotrophic factor, BDNF, has an important role in a variety of neuronal aspects, such as differentiation, maturation, and synaptic function in the central nervous system (CNS). BDNF stimulates mitogen-activated protein kinase/extracellular signal-regulated kinase (MAPK/ERK), phosphoinositide-3kinase (PI3K), and phospholipase C (PLC)-gamma pathways via activation of tropomyosin receptor kinase B (TrkB), a high affinity receptor for BDNF. Evidence has shown significant contributions of these signaling pathways in neurogenesis and synaptic plasticity in in vivo and in vitro experiments. Importantly, it has been demonstrated that dysfunction of the BDNF/TrkB system is involved in the onset of brain diseases, including neurodegenerative and psychiatric disorders. In this review, we discuss actions of BDNF and related signaling molecules on CNS neurons, and their contributions to the pathophysiology of brain diseases.

## 1. Introduction

BDNF, one of the neurotrophic factors, is expressed in a variety of brain regions including the cortex and hippocampus, and exerts beneficial effects in the development, survival, and maintenance of neurons in the central nervous system (CNS). It is well known that MAPK/ERK-, PI3K/Akt, and PLC-gamma signaling pathways are stimulated after activation of TrkB receptor by BDNF, and intensive investigations on these signaling have been performed because the BDNF/TrkB-mediated intracellular signals are involved in many neuronal aspects, including neuronal survival and synaptic plasticity [1,2,3,4]. Increasing evidence demonstrates its contributions in the recovery from depressive symptoms by stimulating neurogenesis in hippocampal region [4,5,6]. Several lines of studies suggest that the BDNF/TrkB system is involved in hippocampal neurogenesis with distinct effects in dentate gyrus (DG) and subventricular zone (SVZ) in the hippocampus [7]. The amount of BDNF in brain tissues is regulated via several cellular process (e.g., transcription, translation, and BDNF mRNA and protein stability). Importantly, BDNF protein is synthetized as a pre-pro form that consists of signal peptide, the pro-region, and the mature BDNF [8,9,10]. The pro-region is removed by proteases intra-/extra-cellularly and secreted mature BDNF binds to TrkB receptor. TrkB receptor possesses tyrosine residues in its kinase domain, and phosphorylated-TrkB triggers to stimulate signaling pathways, including MAPK, PI3K and PLC-gamma cascades, to exert beneficial effects such as neuronal survival promotion. On the other hand, truncated form of TrkB that does not have intracellular kinase domain functions as dominant negative inhibitor against mature BDNF signaling via activation of full length TrkB. Further, it is well-known that pro-BDNF preferentially binds to pan-neurotrophin receptor p75 (p75^NTR^), which causes negative effects such as neuronal death while mature BDNF binds to p75^NTR^ with low affinity [8,9,10]. In this review, we mainly focus on the role of mature BDNF through TrkB receptor in neurogenesis and neurodegeneration.

Concerning neurodegenerative disorders, such as Alzheimer’s disease (AD) that exhibits memory and cognitive deterioration, positive contribution of BDNF in the therapeutic outcomes has been demonstrated because downregulation of neurotrophic signals are considered to be associated with the onset of AD [11]. It has been also reported that downregulation of mRNA [12] and protein [13] of BDNF in the substantia nigra of the postmortem brains of the patients with Parkinson’s disease (PD), one of the progressive neurodegenerative disorders, suggesting that impaired BDNF/TrkB system is involved in the progression of PD symptom.

In this review, we discuss recent findings concerning relationships between the BDNF/TrkB system and neurogenesis, and possible contribution of this system against progression of neurodegenerative disorders. Especially, we introduce small compounds that can directly stimulate TrkB or induce BDNF upregulation because small compounds are expected to penetrate the blood-brain barrier (BBB).

## 2. Activation of BDNF/TrkB System by Compounds

It is well known that BDNF and its high affinity receptor TrkB play essential roles to support survival in a variety of neuronal populations in the CNS and that they contribute to protect neurons against neurodegenerative disease, including AD [14,15,16]. Furthermore, downregulation of BDNF has been reported to be involved in the pathogenesis of mental disorders and neurodegenerative diseases, including AD, PD, and Huntington disease (HD). Therefore, activation of the BDNF/TrkB system is an attractive therapeutic target to treat these brain diseases.

Recently, evidence has demonstrated that induction of BDNF expression via stimulating/modulating activity of neurotransmitter receptors, and application of small molecules that activate TrkB are beneficial strategy for both neuroprotection and neuroregeneration. For example, activating 5-hydroxytryptophan 1A (5-HT1A) or 5-HT2A receptors that induce hippocampal BDNF expression has been examined. Afshar et al. investigated the effects of NAD-299 (an antagonist for 5-HT1A receptor) and TCB-2 (an agonist for 5-HT2A receptor) on learning and memory function in streptozotocin (STZ)-induced memory deficit animal models [17]. In their study, rats received intracerebroventricular (icv) injection of STZ exhibited decreased discrimination index in the novel object recognition test, reduced BDNF, and increased Aβ plaques in the brain, which were reversed by treatment with NAD-299 or TCB-2 [17]. Influence of neuropeptide Y (NPY), which is implicated as potential therapeutic target of HD, in expression of BDNF has been reported [18]. Activation of Y2 receptor (Y2R) by NPY or NPY13-16 (a selective agonist for Y2R) resulted in significant recovery from motor dysfunction in R6/2 mouse, a mouse model of HD. The activation of Y2R also decreased aggregated mutant huntingtin and increased levels of cAMP-regulated phosphoproteins, BDNF, and activated ERK1/2 in the HD model mice while selectively antagonizing Y2R by SF31 blocked the beneficial effects [18]. Recently, using R6/2 mice, Pardo et al. have shown that A-971432, a new selective agonist for sphingosine-1-phosphate (S1P) receptor 5, is a therapeutic candidate for the treatment of HD [19]. They found that chronic administration of A-971432 delayed the disease progression and prolonged the lifespan of symptomatic HD mice. Furthermore, they showed that the beneficial effects of A-971432 included the activation of the BDNF/TrkB system, with a significant decrease in mutant huntingtin aggregation [19].

In addition to stimulation of receptors with neurotransmitters, small compounds also induce upregulation of BDNF. Quercetin (3,5,7,30,40-pentahydroxyflavone), one of the flavonoids found in vegetables and fruits, has been shown protective effects against various diseases, including cancer, cardiovascular diseases, and neurodegenerative disorders [20,21]. Recently, increased expression of BDNF in the hippocampus of male rats that were received daily gavage with quercetin (20 or 50 mg/kg·bwt, for 30 days) has been reported, implying an involvement of BDNF in the neuroprotection by quercetin [22]. 7,8-Dihydroxyflavone (7,8-DHF), another flavonoid, stimulates TrkB receptor and exerts neuroprotective effect against traumatic brain injury [23]. Importantly, 7,8-DHF has a longer half-life time compared to BDNF and is able to pass the BBB due to its small molecular size, suggesting that the flavonoid is useful as a non-invasive clinical drug application [23]. Using HT22 cells, a mouse hippocampal neuronal cell line, positive effects of huperzine A (HupA) against oxidative/glutamate toxicity was reported. Cell death of HT22 cells after glutamate exposure was significantly restored by HupA treatment [24]. Such a HupA treatment reversed the downregulation of pAkt, p-mammalian target of rapamycin (pmTOR), pp70s6 kinase, and protein levels of both BDNF and pTrkB whereas NGF, NT-3, and NT-4 levels were not recovered, suggesting possible involvement of the BDNF/TrkB system in the effect of HupA [24]. In a rat model of acute spinal cord injury (SCI), Jisuikang, Chinese medicine compound, promoted recovery in neurological functions. Using the SCI model, in addition to increased survival rate of neurons, upregulated protein levels of BDNF was observed after Jisuikang administration [25].

It is well known that lipopolysaccharide (LPS), an endotoxin from Gram-negative bacteria, causes inflammation and deficits in learning and memory function in the CNS. Using mice treated with this endotoxin, Chowdhury et al. showed that imperatorin (IMP), one of the phytochemicals, exerted beneficial effects against poor memory retention (the Morris water maze and Y maze tests), via increasing TNF-α and IL-6 levels, and reducing hippocampal/cortical BDNF levels [26]. It has been reported that sulforaphane (SFN) is also effective for recovery from dysfunction of spatial memory caused by LPS [27]. Abnormal behaviors of the mice received LPS injection in the Morris water maze test was reversed by administration of SFN. Notably, an inhibitor for TrkB or mTOR signaling blocked the effects of SFN, suggesting that importance of BDNF/TrkB/mTOR signaling activation in the effect [27]. It has been reported that downregulation of PI3K/Akt/GSK3β signaling caused by sodium arsenite was ameliorated by curcumin application [28]. Rat received sodium arsenite showed decreased expression levels of BDNF, pAkt, pERK, pGSK3β, and p-cAMP response element binding protein (pCREB) in hippocampal region, which were reversed by curcumin application. In addition, curcumin was also effective on the learning and memory deficits in rats caused by sodium arsenite, suggesting that PI3K/Akt/GSK3β signaling stimulated by BDNF is involved in the effects of curcumin [28]. Interestingly, hippocampal Aβ accumulation and cognitive deficits in contextual fear conditioning in mice after repeated peripheral administration of LPS was observed, and the cognitive deficits associated with the increased hippocampal Aβ was reversed by a benzodiazepine agonist MRK-016 [29]. In their system, mRNA levels of BDNF decreased by LPS injections were also restored by MRK-016 [29]. Furthermore, effect of combined zinc and folic acid administration in a depression model established by chronic mild unpredictable stress was also studied [30]. Dou et al. compared the levels of 5-HT, dopamine and norepinephrine in four rat groups; control, paroxetine alone (P), zinc + folic acid (ZnY), and zinc + folic acid+ paroxetine (ZnYP), and found that 5-HT, dopamine and norepinephrine, NMDA, and TrkB mRNA levels in the frontal cortex were significantly increased in the ZnY group while no upregulation of BDNF was observed [30].

## 3. BDNF and Adult Neurogenesis

In several species, including rodents and human, generation of neurons persists throughout the life in some brain regions, such as the hippocampus. Growing evidence indicates that neurogenesis in the adult brain has been implicated in physiological brain function, such as learning and memory [31]. In addition, possible roles of adult neurogenesis in the pathogenesis of brain injury, neurodegenerative and psychiatric diseases have been extensively investigated [32,33]. In the adult rodent brain, neurogenic region is restricted in hippocampal DG and SVZ lining the lateral ventricles [31]. Interestingly, several lines of evidence demonstrate that BDNF plays an important role in adult neurogenesis although its effect is different between DG and SVZ regions [7].

In DG, neural progenitor cells (NPCs) constantly differentiate into excitatory granule neurons which migrate to the granule cell layer and are integrated into hippocampal neural circuits [7]. An in vitro study using neurosphere culture of NPCs isolated from adult rat hippocampus has demonstrated that BDNF promoted cell proliferation, neural differentiation and cell survival [34]. It has also been reported that the neurogenesis-promoting effect of BDNF in organotypic mice hippocampal slice culture was inhibited by pre-incubation with a TrkB antagonist, indicating important contribution of the BDNF/TrkB system [35]. Intrahippocampal infusion of BDNF in adult rats increased the number of newborn neurons and caused appearance of ectopic granule cells, suggesting that BDNF influences not only neurogenesis but also migration of newborn neurons [36]. In order to reveal a contribution of endogenous BDNF, several lines of genetically modified mice targeting BDNF or TrkB were generated. BDNF heterozygous mice (BDNF^+/−^) showed a decreased number of proliferative cells and newborn neurons in DG [37]. Genetic ablation of TrkB in NPCs (but not neurons) caused a reduction of proliferative hippocampal NPCs and newly generated granule neurons [38]. Inconsistent with these reports, another group reported a significant increase of proliferative cells and decrease of newborn neurons in DG in both BDNF ^+/−^ mice and dominant negative TrkB-overexpressing mice [39]. Although the reason of these discrepancies is unclear, consistent results on the reduction of newborn neurons have been highlighted the central role of BDNF in survival of newly generated neurons. Wang and colleagues showed that newborn granule neuron-specific BDNF deletion results in repression of dendrite growth and synaptogenesis to a similar extent of brain-specific BDNF conditional knockout mice [40]. Interestingly, they also found that newborn neuron-targeted *BDNF* gene transfer in brain-specific BDNF knockout mice recovered the deficit in dendrite and synapse in these neurons, demonstrating cell-autonomous actions of BDNF [40]. Such an abnormal dendrite growth and synaptogenesis is also observed in adult NPC-specific TrkB conditional knockout mice [41]. Note that this TrkB-deficient mouse exhibited disruption of neurogenesis-dependent long-term potentiation in DG, supporting the neurogenic effect of BDNF/TrkB signaling in the hippocampal circuits.

In SVZ regions, NPCs continuously differentiate into neuroblasts, and migrate into the granular cell layer or glomerular layers of olfactory bulb (OB), then differentiate into inhibitory interneurons or dopaminergic neurons [7]. Unlike NPCs in DG region, NPCs in SVZ and neuroblasts do not express TrkB although a few of them express pan-neurotrophin receptor p75^NTR^ [42]. Young and colleagues reported that 0.3% of cells in SVZ region express p75^NTR^ and these cells have ability to form neurosphere in vitro [42]. BDNF treatment to the neurospheres derived from p75^NTR^-positive cells increased neural production and this effect was disappeared in neurospheres derived from p75^NTR^-deficient mice [42]. The p75^NTR^-deficient mice have a reduced number of SVZ neuroblasts in vivo, suggesting that p75^NTR^ signaling is essential for SVZ neurogenesis [42]. However, BDNF action on NPCs in SVZ and neuroblasts is still controversial because some group reported that infusion or overexpression of BDNF in SVZ region did not affect the number of newborn OB neurons whereas other group showed that intraventricular BDNF application increased the number of newly-generated OB neurons [43,44,45]. Thus, further study is required to clarify the contribution of BDNF on SVZ neurogenesis. After the migration of neuroblasts into the OB, these cells began to differentiate into neurons and express TrkB [46]. Similar to hippocampal DG, newborn neuron-targeted deletion of TrkB resulted in an impairment of dendritogenesis and spine formation in the OB newborn neurons, suggesting conserved role of the BDNF/TrkB system in neural maturation [46]. Furthermore, TrkB deletion severely decreased the survival of newborn dopaminergic neurons but not interneurons in glomerular layers [43]. Interestingly, the specific loss of dopaminergic neurons was mirrored by a corresponding increase in the number of interneurons, highlighting essential roles of TrkB in balancing the incorporation of defined classes of adult-born neurons in the OB [43].

## 4. Monoaminergic System and BDNF-Mediated Neurogenesis

**Figure 1:**
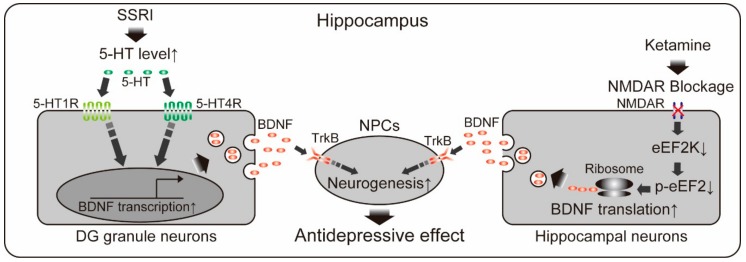
Schematic illustration of a possible molecular mechanism of selective serotonin reuptake inhibitor (SSRI)- or ketamine-induced antidepressive effect. NCPS: neural progenitor cells; DG: dentate gyrus. Both antidepressants increase BDNF production in hippocampal neurons via distinct pathways and finally increased neurogenesis by stimulating TrkB in NPCs. ↑:Upregulation; ↓:Downregulation; × at NMDAR means blockage of NMDAR; → at BDNF transcription means transcription of BDNF genes.

For over a decade, roles of neurogenic action of BDNF in the antidepressive effects of pharmacological and physical therapies have been well documented. Most of currently available antidepressants are categorized in monoaminergic antidepressants which primary act via increasing the activity of monoaminergic (serotonin, and norepinephrine) neural circuits usually within a few hours. In spite of the rapid increase of monoamine levels induced by these drugs, chronic administration for several weeks is required to achieve therapeutic response in patients with depression [47]. One of the possible explanations in this time-course discrepancy is a delayed induction of hippocampal neurogenesis by antidepressants. Chronic, but not acute, antidepressant treatment increases the number of proliferative cells and newborn neurons in rodents, which is consistent with the time-course of the antidepressive effect [48,49]. Investigation of post-mortal brain of depressive patients who had been treated with antidepressants also showed neurogenic action of antidepressants in the hippocampus [50,51,52]. Ablation of NPCs in adult DG by irradiation diminished antidepressant-induced antidepressive behavior in mice, demonstrating a requirement of hippocampal neurogenesis for the behavioral effect of antidepressants [48]. As expected, accumulating evidence indicates that antidepressant-induced neurogenic action is mediated by BDNF. It have been consistently reported that BDNF expression in the hippocampus after chronic (not acute) antidepressant treatment was increased in rodents and human [53,54,55]. Causative role of BDNF/TrkB signaling in the antidepressant-induced hippocampal neurogenesis was examined using genetic deletion of TrkB in hippocampal NPCs [38]. Conditional TrkB-deficient mice did not show both antidepressant-induced neurogenic effect and antidepressive behavior. Moreover, BDNF injection into hippocampal DG produced antidepressive effect as early as 3 days after its single administration and lasted for at least 10 days [56]. As BDNF infusion directly induces hippocampal neurogenesis, this antidepressive effect of BDNF may also be mediated by enhancement of neurogenesis [36]. Recent studies have addressed how antidepressants increase levels of BDNF and neurogenesis. Samuels and colleagues reported that genetic deletion of 5-HT1AR in DG granule neurons abolished antidepressive behaviors induced by selective serotonin reuptake inhibitors (SSRIs, widely used classes of antidepressants) and attenuated increased neurogenesis and BDNF [57]. Notably, they also found that specific deletion of 5-HT1AR in newborn granule cells in DG did not impair SSRI-induced behavioral and neurogenic responses, suggesting that 5-HT1AR signaling non-autonomously affects neurogenic responses. In addition to 5-HT1AR, contribution of 5-HT4R has also been reported. Pharmacological inhibition of 5-HT4R diminished antidepressive effects of SSRIs and 5-HT4R stimulation conversely exhibited antidepressant effects [58]. Imoto and colleagues showed that 5-HT4R knockout mice failed to respond to SSRI [59]. Interestingly, they found that chronic SSRI treatment decreased a maturity of preexisting granule neurons in DG (called “dematuration”) and this dematuration was not observed in 5-HT4R knockout mice. As BDNF induction and neurogenic action by SSRI were well correlated with the progress of dematuraion of granule neurons, the reduced maturity of granule neurons may be accountable for SSRI action although further studies to elucidate causal role of the dematuration by SSRI in neurogenesis may be required. Overall, current knowledge of SSRI actions in DG suggests that increased serotonin stimulates 5-HT1AR and/or 5-HT4R in preexisting granule neurons to induce neurogenesis via non-cell autonomous mechanism, possibly through enhanced production and secretion of BDNF (Figure 1).

## 5. Other Antidepressive Therapy and BDNF-Mediated Neurogenesis

Involvement of BDNF/TrkB signaling and hippocampal neurogenesis have also studied in not only monoaminergic antidepressants therapy but also in other types of antidepressive therapies including electroconvulsive shocks, exercise, enriched environment, flavonoids, and non-conventional antidepressants treatment [38,60,61,62,63,64,65]. It has been demonstrated that these therapies stimulated both the BDNF/TrkB system and hippocampal neurogenesis [38,60,61,62,63,64,65]. In addition, causative role of BDNF-mediated neurogenesis in antidepressive effects have also been demonstrated. NPC-specific TrkB knockout mice were insensitive to the exercise-induced improvement in depressive behaviors and showed no change in the number of proliferative cells and newborn neurons in DG after exercise [38]. Antidepressive effect and neurogenic action of silibinin, a polyphenolic flavonoid from *Silybum marianum*, was abrogated by icv injection of a TrkB antagonist in mice [63]. Recently, ketamine, an NMDA receptor antagonist, has been attracting researcher’s attention as a non-conventional antidepressant [66]. Single administration of ketamine produces rapid-onset antidepressant response in depressive patients within few hours (lasting 1–2 weeks) unlike effects by monoaminergic antidepressants. Therapy-response rate reaches over 60% of monoaminergic antidepressant drug non-responders and tolerability seems acceptable at least in the short term [66]. Recent animal studies revealed an involvement of BDNF and neurogenesis in the ketamine-induced antidepressive effects [64,65]. NMDA receptor blockage by ketamine led to deactivation of eukaryotic elongation factor 2 (eEF2) kinase, which resulted in decreased eEF2 phosphorylation and de-suppression of BDNF expression within 30 min after treatment [64]. BDNF heterozygous mice did not show antidepressive behavior at the time, suggesting rapid requirement of BDNF in the antidepressive effect of ketamine [64]. Increased hippocampal neurogenesis was also observed in the ketamine-treated mice at 24 h after treatment [65]. Deletion of TrkB in adult NPCs diminished the neurogenic effect of ketamine and antidepressive behaviors at 24 h after the treatment. Note that antidepressive behaviors at 1 h after ketamine treatment was not affected by adult NPCs-specific TrkB deletion, suggesting that the sustained antidepressive effect of ketamine depends on BDNF/TrkB-mediated neurogenesis while rapid antidepressive effect is occurred through neurogenesis-independent BDNF/TrkB action [65].

Although possible contributions of BDNF-mediated neurogenesis in the antidepressive therapy is likely, BDNF delivery into hippocampus is very difficult because of its inability to cross the BBB and short half-life time (<10min) [23]. Another option is to use other growth factors that have similar biological effects as BDNF. For example, insulin growth factor I (IGF-I) is one of the candidates because IGF-I can stimulate the same intracellular signaling as BDNF via activating the IGF-I receptor and penetrate the BBB [67]. It has been reported that IGF-I treatment enhances proliferation and differentiation of NPCs in vitro [68,69,70]. Recently, we reported that IGF-I application restored impairments in differentiation/maturation of newborn neurons caused by exposure to corticosterone, a stress-related hormone, in vitro [71]. Neurogenesis induced by IGF-I in adult hippocampal DG was revealed by in vivo studies using both subcutaneous (sc) and icv injection [72,73,74]. Furthermore, several animal studies indicate that icv injection of IGF-I produces antidepressive behavior in rodents [75,76]. However, requirement of neurogenic action in antidepressive effects by IGF-I is controversial because one study reported no change in neurogenesis when antidepressive behavior was appeared [75]. Therefore, precise research using irradiation technique to kill NPCs or adult NPC-specific IGF1R knockout mice is useful to clarify the relationship between neurogenesis and antidepressive behavior. It is also important to examine whether sc injection of IGF-I can achieve antidepressive effects. Although further studies were required, IGF-I has a potential to ameliorate depressive symptoms via non-conventional strategies.

## 6. Influence of Val66Met Polymorphism of BDNF on the Neurogenesis

Recently, many researchers have focused on possible relationship between BDNF polymorphism and brain disease including neuropsychiatric disorders [77]. A non-synonymous polymorphism which causes a valine (Val) to methionine (Met) substitution at position 66 of BDNF genes is carried by 40–50% and 25–32% of the Asian and Caucasian population, respectively [78]. Remarkably, several genetic studies have showed that Met carriers displayed smaller hippocampal volumes and lower recognition memory performance compared with Val homozygotes [79,80,81,82]. In vitro studies using mouse neurons reported that the Val66Met polymorphism impaired trafficking of BDNF mRNA to dendrites, localization of BDNF in secretory granules and activity-dependent secretion of BDNF [81,83]. In order to investigate possible impact of the Val66Met polymorphism on brain functions and behaviors, several animal studies using a variant BDNF knock-in mouse carrying BDNF^Met/Met^ have been also reported. BDNF^Met/Met^ mice exhibited defects in survival but not proliferation of adult-born neurons in both hippocampal DG and SVZ [84,85]. These mice exhibited increased anxiety-related behaviors when placed in stressful condition, and these behavioral abnormalities were not rescued by the treatment of a SSRI, fluoxetine [86]. It has been reported that chronic fluoxetine treatment failed to increase levels of BDNF, survival of newborn neurons, long-term potentiation in DG, and antidepressive behaviors in BDNF^Met/Met^ mice [84]. Recently, it has been also reported that BDNF^Met/Met^ mice did not show exercise-induced upregulation of BDNF, increase of newborn neurons, and antidepressive effects [87]. In addition to neurogenesis in the DG, dendritogenesis and synaptogenesis in the prefrontal cortex were also impaired in BDNF^Met/Met^ mice [88]. BDNF Val66Met polymorphism caused constitutive atrophy of distal apical dendrites, decreased spine density/diameter and decrements in excitatory postsynaptic currents in layer V pyramidal neurons. Moreover, the ketamine-induced synaptogenesis and antidepressive behavior were also blocked in the mice [88]. It is possible that antidepressive effects induced by antidepressants and physical exercise are affected by alteration in the activity-dependent BDNF secretion due to Val66Met polymorphism.

## 7. Alzheimer’s Disease and BDNF

AD is a progressive neurodegenerative disorder that is accompanied by symptoms, such as memory and cognitive deterioration. Patients with AD show several neuropathological features, including extracellular amyloid plaques composed of aggregated Aβ peptide and intracellular neurofibrillary tangles of aggregates consisting of abnormally phosphorylated tau, a soluble microtubule-associated cytoskeletal protein. Degeneration especially in cholinergic neurons in basal forebrain regions is considered as the earliest pathological event in AD. Because these neurons innervate a variety of brain regions, including the hippocampus and cortex, a reduction in the retrograde neurotrophic signals are considered to be involved in the onset of AD [11].

Aβ peptides of either 40 or 42 amino acids are produced in the process of aberrant proteolytic cleavage of amyloid precursor protein (APP) by β- and γ-secretases [89]. Although α-secretase also cleaves APP effectively, the cleavage is occurred within the Aβ sequence and does not produce Aβ peptides. As the result of altered balance in the APP processing, the over-produced Aβ peptides aggregate to form soluble oligomers or insoluble beta-sheet fibrils accumulated in diffuse senile plaques, likely depending on the extracellular concentration of Aβ peptides [90]. Although it is still unclear how hyperphospholyration of tau is caused, there is a reciprocal relationship between Aβ peptides and tau hyperphospholyration and aggregation. Oxidative stress by Aβ oligomers was able to stimulate tau hyperphosphorylation [91,92] while site-specific phosphorylation of tau can protect neurons from Aβ toxiciticy [93].

A glycoprotein ApoE is also known as one of the risk factors for AD. It mediates the delivery of lipids like cholesterol via ApoE receptors in the membrane. Human ApoE proteins consist of three isoforms ApoE2, E3, and E4, with different isoelectric point as pH 5.4, 5.5, and 6.1, respectively [94]. There is no consensus on how only ApoE4 contributes to the pathogenesis of AD. However, a recent study showed that ApoE secreted from glial cells triggered the transcription of APP in neurons through MAPK pathway and AP-1 (activator protein 1), a transcription factor. The Aβ production induced by glial ApoE4 was most potent among the three types of ApoE [95].

Due to the essential roles in neuronal aspects of CNS function as mentioned above, possible contribution of the BDNF/TrkB dysfunction in the development of AD has been demonstrated. Interestingly, some reports showed reduced levels of BDNF and TrkB in patients with AD while others demonstrated the opposite results. Ferrer and colleagues reported decreased BDNF and full-length TrkB levels in the frontal cortex of patients with AD, and increased truncated TrkB that lacks its intracellular kinase domain and is mainly expressed in glial cells [96]. Downregulation of the BDNF transcripts in parietal cortex [97,98] and reduced protein levels of BDNF in the hippocampus and parietal cortex [99,100] have also been reported. Recent study showed a negative correlation between BDNF mRNA expression in the dorsal lateral prefrontal cortex and cognitive decline in the AD patients [101]. On the other hand, increased plasma concentration of BDNF has been observed in AD and mild cognitive impairment patients [102,103]. Similarly, it is suggested that the levels of BDNF are inconsistently regulated by Aβ in the preclinical studies. Xia et al. demonstrated that BDNF expression in the cerebral cortex and hippocampus was markedly reduced in an AD mouse model (APP/PS1 transgenic mice) at 3 and 9 months old [104]. Icv injections of Aβ oligomers to 3-month-old C57BL/6 mice dramatically decreased BDNF protein and mRNA levels within 24 h. They further clarified that peroxisome proliferators-activated receptor γ coactivator 1 α (PGC-1α) and fibronectin type III domain-containing 5 (FNDC5) were involved in the Aβ-dependent downregulation of BDNF [104]. In contrast, elevated BDNF protein and mRNA levels were observed in SH-SY5Y human neuroblastoma cells after exposure to Aβ peptides [105].

It has been reported that neuronal cell death and dysfunction in the animal models of AD were ameliorated through activating BDNF/TrkB signaling. Lentiviral BDNF gene delivery into the entorhinal cortex of transgenic mice expressing the APP with mutations at age 2 months improved hippocampal-dependent contextual fear conditioning after 3 months [106]. Obvious cell loss observed in the layers II-VI of the entorhinal cortex of these transgenic mice was also ameliorated by the BDNF delivery. Interestingly, the BDNF gene delivery did not change the number of amyloid plaque, indicating that the neuroprotective effect of BDNF was not through the downregulation of Aβ in the AD models [106]. Rutin (3,3’,4’,5,7-pentahydroxyflavone-3-rhamnoglucoside), one of the flavonoids, rescued reduction in the memory retrieval function in rats injected Aβ into the deep frontal cortex, through upregulation of BDNF and MAPK pathway activation [107]. Similarly, a 5 consecutive day intraperitoneal (ip) administration of curcumin, a major polyphenol from *Curcumalonga*, ameliorated cognitive deficits in rats induced by an icv injection of Aβ, via the upregulation of BDNF and activating MAPK pathway. Sole injection of BDNF into the hippocampus also improved the impairment in cognitive function [108]. Oral administration of SCM-198, an alkaloid extracted from *Herba leonuri*, for 3 months improved both recognition and spatial memory in the APP/PS1 transgenic mice without affecting the amount of Aβ. Importantly, SCM-198 ameliorated the decline in BDNF expression and TrkB activation both in in vivo and in cortical cultures [109].

Low-level laser therapy, a recently developed intervention, regulates neuronal function in neuronal cultures, animals, and clinical conditions [110] and rescues the neuronal loss and dendritic atrophy in cultured hippocampal neurons treated with Aβ and hippocampal neurons from the APP/PS1 mice via increasing BDNF expression [111]. As mentioned above, there is accumulating evidence for a potential use of 7,8-DHF, a small-molecule of TrkB agonist, in the therapeutic effect for AD patients. Importantly, 7,8-DHF can penetrate the BBB and systemic administrations of 7,8-DHF for 10 consecutive days restored the memory deficits of 5XFAD mice model of AD. 7,8-DHF blocked β-secretase elevation that causes Aβ accumulation in the 5XFAD mouse brain [112]. Furthermore, 7,8-DHF prevented the loss of hippocampal synapses and memory deficits in the 5XFAD mice [113,114,115].

Voluntary exercise that has been reported to increase BDNF in the several brain regions [116] was tested in the AD models. As expected, treadmill exercise saved neurons from Aβ-induced cell death in the cortex of the APP/PS1 mouse [117] and improved the spatial memory function of another AD model mouse, in both of which BDNF upregulation was confirmed to be involved in the effects [118]. However, there is no significant change in the accumulation of Aβ in either the cerebral cortex or hippocampus after exercise. It was also reported that BDNF reduced production of Aβ peptides through enhanced α-secretase-mediated processing of APP in the APP/PS1 transgenic mice [119]. The relationship between BDNF and APP/Aβ seems to be more complex. The pro-domain of BDNF protein exerted toxic effects only in the presence of Aβ in SH-SY5Y human neuroblastoma cells [120]. Ruiz-Leon et al. demonstrated that BDNF stimulated APP expression by activating promoter of *APP* gene through TrkB-, Ras/MAPK- and PI3K/Akt-dependent mechanisms in SH-SY5Y cells [121]. It was also reported that ApoE4 carriers have reduced serum BDNF levels [122]. Sen and colleagues showed that ApoE4 increased nuclear translocation of histone deacetylase 6 (HDAC6) in human neurons, which in turn suppressed BDNF transcription from the BDNF exon IV by binding of HDAC6 to the promoter region. In contrast, ApoE3 increased acetylation of histone 3 and stimulated BDNF expression [123]. They further reported regulatory roles of ApoE in BDNF secretion from cultured astrocytes. ApoE2 treatment for 24 h increased intracellular amount of pre-pro BDNF while ApoE4 reduced it. Astrocytes exposed to ApoE3 secreted 1.75-fold higher pro-BDNF than ApoE2-treated astrocytes. Furthermore, ApoE2-treated astrocytes secreted mature-BDNF 3 times higher than ApoE3-treated astrocytes. ApoE4 dramatically reduced secretion of both forms of BDNF from astrocytes [124]. These data shows reciprocal and complex interactions between BDNF/TrkB signals and pathogenesis of AD.

On the other hand, several studies indicated that phosphorylation of tau protein is also regulated by the BDNF/TrkB system. Elliott et al. reported that BDNF induced a dephosphorylation of tau in P19 mouse embryonic carcinoma cells within 15 min via PI3K signaling pathway [125]. 7,8-DHF was also reported to inhibit expression of abnormal tau via activating PI3K/Akt pathway [126]. MiR-322, the rodent homologue of human miR-424, is predicted to target the 3’-untranslated region (3’-UTR) of BDNF mRNA and found to be increased in AD mouse brain. Zhang et al. recently showed that overexpressed miR-322 promoted tau phosphorylation by negatively regulating BDNF levels and TrkB activation whereas silencing miR-322 increased TrkB activation and accelerated dephosphorylation of tau in Neuro2A mouse neuroblastoma cells [127]. Intralateral ventricle injection of adeno-associated virus carrying *BDNF* gene in P301L mouse model of tauopathy at 3 months of age before the occurrence of neurofibrillary tangle formation in the mice prevented the neuronal loss and the behavioral deficits without affect hyperphosphorylation levels of tau [128]. There are several studies that investigated the effect of abnormal tau on expression and secretion of BDNF. In a mouse model of tauopathy, P301S tau transgenic (P301S) mice show memory deficits, synaptic and neuronal loss in the cortex and spinal cord due to the fibrillary tau tangles emerged throughout the CNS [129]. Rosa and colleagues reported a secretion of BDNF was impaired in the vitreous of the mouse model while mRNA and protein levels of BDNF in retinas were not altered [130]. Both BDNF expression and secretion in rat primary cortical neurons were significantly suppressed by okadaic acid-induced tau hyperphosphorylation [131]. Although these findings suggest a limitation of the usage of serum BDNF levels as a biomarker of AD for early diagnosis, potentiation of BDNF/TrkB signals will be a one of the therapeutic targets for the treatment of AD symptoms. Future studies on other small molecules and the gene delivery, and cellular transplantation that stimulate BDNF/TrkB signaling pathways are important.

## 8. Parkinson’s Disease and BDNF

Parkinson’s disease (PD) is also progressive neurodegenerative disorder like AD. PD is clinically characterized by bradykinesia, rigidity and rest tremor, and is pathologically characterized by the loss of dopaminergic neurons in the substantia nigra pars compacta, which results in the decline of dopamine (DA) levels in the striatum. Dystrophic neurites and intracellular inclusions called “Lewy bodies” which mainly contain α-synuclein are also pathological hallmarks of PD [132,133]. It is well known that patients with PD also display mild cognitive deficits correlated with severity of symptoms. Because BDNF is an important mediator for neuronal survival and synaptic plasticity to maintain brain functions, possible contribution of BDNF/TrkB dysfunction in the cognitive deficits in PD patients was hypothesized. Positive correlations between serum BDNF concentration and severity of the symptoms [134,135] and cognitive deficits [136,137] in the patients with PD have been reported. Reduced levels of BDNF mRNA [12] and protein [13] in the substantia nigra of the postmortem PD patients were also demonstrated. Furthermore, serum BDNF levels was negatively correlated with degeneration levels of striatal dopaminergic neurons [138]. However, a large-scale study in which over 600 individuals were participated concluded that significant decrease of serum BDNF levels were observed in patients with AD, frontotemporal dementia, Lewy body dementia, vascular dementia whereas significant increase was confirmed in PD patients [139]. On the other hand, the latest study of the meta-analysis indeed supports an association between decreased blood concentration of BDNF and PD [140].

Preclinical studies using animal models of PD have been carried out to investigate whether BDNF dysfunction is linked to the PD symptoms. MPTP (1-methyl-4-phenyl-1,2,3,6-tetrahydropyridine), a dopamine neurotoxin, induces parkinsonism in animals. Using female rhesus monkeys received MPTP administration, Collier et al. found a decline of BDNF levels in striatal tissue of young and middle-aged animals compared with age-controlled animals while no significant change in the tissue of old animals where baseline BDNF levels were decreased [141]. Another model of preclinical stage of PD induced by 6-hydroxydopamine (6-OHDA) which causes moderate dopaminergic lesion has been examined. Bilateral injections of 6-OHDA into the rat ventral region of the caudate-putamen caused downregulation of BDNF and TrkB mRNA levels in the hippocampus, amygdala, and habenula [142]. Rats received 6-OHDA administration in the striatum exhibited reduction of BDNF but increase of TrkB [143].

Supporting the idea that BDNF dysfunction is involved in the PD symptoms, clinical rehabilitations and treatments likely ameliorates decline of BDNF in PD patients and animal models. A protocol of cognitive rehabilitation such as a 1-month 12-session treatment (three sessions a week) which focuses on the training of executive functioning increased BDNF levels in serum of PD patients affected by mild cognitive impairment, with improved cognitive performance [144,145]. Enhanced activation of the BDNF/TrkB system in lymphocyte after an intensive rehabilitation in PD patients was also reported [146]. Recent reports also showed that exercise or physical training ameliorated the reduced levels of BDNF in patients with PD [147]. In the animal model of PD, 6-OHDA-induced reductions of BDNF and TrkB in the striatum and hippocampus of mice were restored by physical training such as running on a treadmill [148]. The involvement of BDNF/TrkB signals in the ameliorating effect of physical exercise in the PD model was confirmed by using K252a, a blocker for Trk receptors [149]. There are several studies that show a protective effect of 7,8-DHF in degeneration of dopaminergic neurons through TrkB activation in the rodent models of PD induced by 6-OHDA or MPTP [150,151].

Recently, researchers are also focusing on the relationship between BDNF/TrkB functions and α-synuclein in the context of the development of PD. Goldberg and colleagues showed that transplantation of NPCs into the striatum of α-synuclein-expressing mouse model of PD that exhibits progressive development of Lewy body pathology with significant cognitive dysfunction significantly improved cognitive and motor functions. Importantly, transplantation of BDNF-depleted NPCs failed to improve these dysfunctions, and only BDNF gene delivery into the striatum mimicked the effects [152]. Overexpression of α-synuclein in PC12 cells led to suppression of BDNF transcription likely via downregulation of CREB [153]. Kang et al. found an interesting phenomenon that α-synuclein binds directly to the kinase domain of TrkB receptor. The interaction inhibited BDNF/TrkB signaling pathways and caused degeneration of dopaminergic neurons. TrkB ubiquitination was also induced by α-synuclein, which led a reduction in TrkB protein levels [154].

## 9. Huntington’s Disease and BDNF

Huntington’s disease (HD) is also a progressive neurodegenerative disease, characterized by abnormal involuntary movements and cognitive disability [155,156,157]. Patients with HD carry an expanded CAG-repeat in huntingtin (*HTT*) gene that generates abnormally elongated poly glutamine stretch in the N-terminal region of the huntingtin (htt) protein. Because progressive loss of striatal neurons is the most obvious pathological change in HD and these neurons can not produce BDNF, the impaired supply of BDNF from cortical neurons projecting their axons to the striatum is considered to be involved in the neuronal loss observed in HD patients.

Ferrer et al. reported reduced levels of BDNF in the caudate and putamen in the post-mortem brain of HD patients and normal levels in the cerebral cortex and hippocampus [155]. On the other hand, Zuccato et al. showed a significant reduction in BDNF mRNA and protein in the cortex of HD patients by using a systematic and quantitative assessment [156]. Many reports also have shown significant downregulation of BDNF in the several brain regions in animal models of HD [157]. Although correlations between serum levels of BDNF and HD have been controversial, a recent report concluded that levels of BDNF mRNA/protein in human blood samples were not suitable for biomarkers of HD [158]. The mutant htt protein has been considered to be toxic and cause neuronal death in striatal neurons. Recent findings, however, shed light on a beneficial function of wild-type htt protein. Cattaneo and colleagues demonstrated that wild-type htt stimulated transcription of BDNF in cortical neurons, which was critical for the survival of striatal neurons [159]. Conversely, Ma et al. showed normal pro-form, but reduced mature BDNF protein expression and activation of TrkB in the sensorimotor cortex and striatum of an aged HD mouse model, suggesting unaffected BDNF production in the cortical neurons [160]. Normal protein levels of BDNF and TrkB were also observed in another HD mouse, but levels of activated TrkB was reduced by unknown mechanisms in the model [161] while Plotkin et al. reported normal activation of TrkB but decreased downstream signals of TrkB via p75^NTR^-dependent mechanism [162].

Potential therapeutic effects of BDNF have been reported in rodent HD models. Bilateral expression of BDNF by adeno-associated virus in the striatum ameliorated the impairments in both motor and cognitive functions in transgenic HD rats, with increased volume of the striatum and the number of neural cells [163]. Improved motor function in quinolinic acid-induce HD mouse model was also accomplished by transplantation with embryonic stem cell-derived neural progenitors that overexpress BDNF [164]. These results suggest that at last in part of the cause of neuronal loss observed in the striatum of the HD patients could be attributed to the impairment of the BDNF/TrkB signals in the region and compensating these functions would be a promising strategy for the future treatment for HD.

## 10. BDNF/TrkB Transport As a Therapeutic Target of Neurodegenerative Diseases

Characterization of the relationship between BDNF and disease symptoms is difficult as there are several cellular processes (e.g., transcription, translation, and BDNF mRNA and protein stability) to regulate the amount of BDNF in tissues. Given that both reduced and elevated levels of BDNF/TrkB have been reported in the patients and animal models of neurodegenerative disorders, researchers in this field have been asked to find more reliable and direct impairments of the BDNF/TrkB functions in the pathogenesis of the diseases. Possible candidate of the BDNF/TrkB dysfunctions is the intracellular transport process of “synthesized” BDNF-containing vesicles for preparing secretion to surrounding cells and “received” BDNF-TrkB complex to send signals to the cell body.

**Figure 2:**
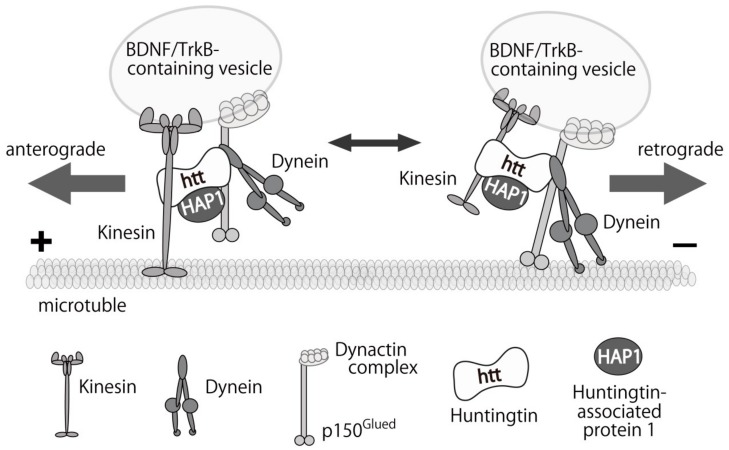
A proposed model of the bi-directional transport of BDNF/TrkB-containing vesicle on microtubules. The motor complex includes the motor proteins, huntingtin, huntingtin-associated protein 1 (HAP1), and dynactin complex. It can involve both kinesin and dynein simultaneously that are responsible for the anterograde (towards to the plus-end of microtubule) and the retrograde (to the minus-end) transport of cargos, respectively. Phosphorylation of huntingtin at serine 421 is possible to regulate the direction of transport, by stabilizing kinesin at the complex and dominantly move to the plus-end. Dynein-dependent retrograde transport becomes dominant when huntingtin is dephosphorylated (See also [166]). Because impaired intracellular transport of BDNF/TrkB-containing vesicles has been suggested as pathophysiology of neurodegenerative diseases, ameliorating the impairment would be a potential therapeutic target for the future medical treatment. +: plus end; -: minus end.

Gauthier et al. revealed an important function of normal htt as a member of motor protein complex [165]. They showed that htt protein positively regulated the axonal transport of BDNF-containing vesicles in cortical neurons, and mutant htt expression or knockdown of normal htt hampered the transport. This report suggested that impaired trophic support from cortical neuron might contribute to the neuronal loss in the striatum, the main pathological change in HD [165]. The transport of BDNF/TrkB-containing vesicles is carried out by kinesin and dynein motor protein complex in a microtubule-dependent movement. The complex consists of normal htt, and huntingtin-associated protein (HAP), dynactin, and Rab GTPase family proteins [166] (Figure 2).

Using a mouse model of AD, Tg2576 transgenic mice, that express the Swedish mutation of the human APP, Poon et al. found significant reduction in the retrograde transport rate of BDNF, and suppression of TrkB signals in cultured cortical neurons. Importantly, they also confirmed that γ-secretase inhibitor restored the reduced BDNF transport and Aβ oligomers alone impaired the retrograde transport of BDNF in axon [167]. Seifert and colleagues also reported impairments in both the anterograde and retrograde transport of BDNF-containing vesicles in hippocampal neurons derived from another AD mouse model (5xFAD) with increased Aβ expression. In the study, a fast action (within 5 min) of Aβ on the transport was also revealed [168]. Gan et al. proposed Ca^2+^-dependent mechanisms of the disruption of BDNF transport induced by Aβ oligomers. Aβ enhanced Ca^2+^ influx via both axonal voltage-gated Ca^2+^ channels and dendritic glutamate receptors. Then, calmodulin binds to free Ca^2+^ and subsequently activates calcineurin-GSK3β, which may in turn phosphorylated motor proteins and disrupt BDNF transport [169]. Interestingly, similar defects in intracellular BDNF trafficking have been reported in PD models. Cultured hippocampal neurons exposed to α-synuclein aggregates disrupted axonal transport of BDNF/TrkB and Rab7-containing endosomes. In addition, TrkB/Rab7 and pERK5 which is responsible for propagation of the TrkB signaling were accumulated with α-synuclein aggregates [170]. Fang et al. showed the similar result that the retrograde axonal transport of BDNF in cortical neurons was dramatically impaired in an α-synuclein transgenic mice [171]. They found a direct interaction of α-synuclein with dynein, which inhibited the trafficking and BDNF/Trk-induced trophic signaling likely through activating Rab5 and Rab7 [171]. Furthermore, immunoreactivity of kinesin was significantly reduced in nigral neurons in the sporadic PD patients, and overexpression of α-synuclein in a PD mouse model resulted in reduced levels of motor proteins, including kinesin and dynein. Importantly, the reduction in kinesin levels preceded the degenerations in dopaminergic neurons, a phenotypic marker for the early stage of PD [172]. These results clearly suggest that preventing the impairments in the intracellular transport of BDNF/TrkB is a possible therapeutic target in these diseases. Recently, we reported a beneficial aspect of glucocorticoid to enhance the transport of BDNF-containing vesicles in cortical neurons. A synthetic glucocorticoid dexamethasone DEX, a potent agonist for glucocorticoid receptor (GR), increased huntingtin protein which forms motor complex with both kinesin and dynein and enhances the microtubule-dependent vesicular transport [173]. Supporting this, offspring from DEX-treated mothers were improved in their memory functions in a mouse model of AD [174], and DEX administration significantly ameliorated the degeneration of dopaminergic neurons in the substantia nigra in the MPTP-induced PD mouse [175]. Although it is possible that an inhibition of inflammatory activity by DEX was involved in these effects, restoring an impaired BDNF transport in model mice is also possible mechanism in the DEX effect. Because BDNF itself cannot cross the BBB, further studies focusing on the intracellular BDNF/TrkB transport would be important for the better understanding of the pathophysiology of neurodegenerative diseases and developing new medical treatments in the future.

## 11. Conclusion

In this review, we introduced the contribution of altered BDNF/TrkB system in the pathophysiology of brain diseases including mental disorders and neurodegenerative diseases. We discussed possible involvement of BDNF upregulation in the improvement of pathophysiological behaviors in disease models. In the process of BDNF upregulation, there are many steps, including transcription, translation, and degradation of both BDNF mRNA and protein. Thus, although characterization of the relationship among BDNF and disease symptoms is indeed difficult due to the multiple processes to regulate the amount of BDNF in tissues, detailed mechanisms underlying the upregulation of BDNF are need to be clarified to achieve more effective therapeutic approaches for neuroprotection. In addition to the cellular processes that regulate the amount of both BDNF mRNA and protein, the changes in the efficiency of secretion and transport of BDNF protein to the secretion sites in neurons may affect synaptic function and cell survival in the pathophysiology. Therefore, although studies concerning small compounds that can increase mRNA and/or protein expression levels of BDNF and regulate the related intracellular signaling molecules have been extensively carried out as mentioned above, to investigate secretion and/or transport of BDNF protein in the presence of these small compounds (e.g., a variety of flavonoid) is very important in the future study.
